# Risk Factors and Laboratory Findings Associated With Diabetic Ketoacidosis in Hospitalized Pediatric Patients

**DOI:** 10.7759/cureus.25410

**Published:** 2022-05-27

**Authors:** Mukul Sehgal, Mansi Batra, Prashant Jha, Omar Sanchez

**Affiliations:** 1 Critical Care Medicine, University of South Alabama, Alabama, USA; 2 Pediatrics, University of South Alabama College of Medicine, Mobile, USA; 3 Pediatric Critical Care Medicine, University of Nevada Las Vegas School of Medicine, Las Vegas, USA; 4 Pediatric Critical Care Medicine, University of South Alabama College of Medicine, Mobile, USA

**Keywords:** pediatric intensive care unit (picu), mean platelet volume (mpv), global cerebral edema, ph, acidosis, oxidative stress, diabetic ketoacidosis (dka)

## Abstract

Background: Diabetic ketoacidosis (DKA), the most serious and acute complication of type 1 diabetes, has an incidence of 6%-8% among known pediatric type 1 diabetes patients, although risk factors associated with severe DKA in the pediatric population are poorly understood [1].

Method: A single-institution, retrospective chart analysis of pediatric DKA patients admitted to our pediatric intensive care unit (PICU) was conducted in South Alabama between October 2017 and April 2021. Laboratory findings were obtained from venous samples collected from the patients on admission.

Results: Of 429 admissions, 256 unique patients were admitted with DKA to PICU during the 3.5-year period; 55.9% of them were males. The median (IQR) age of the patients was 12 (10-15) years, and their median HbA1c level was 11.02 (10%-12%), which was similar to Medicaid and private insurance statistics (11.1 [9.87-12.2] vs 11 [9.65-12], p = 0.4). Serum pH on presentation was 7.17 (7.08-7.25), and serum bicarbonate was 10 (7-14) mmol/L. White blood cell (WBC) count, platelet count, and mean platelet volume (MPV) had a negative correlation with serum pH (r = -0.52, p < 0.001, r = -0.25, p = 0.01 and r = -0.11, p = 0.03, respectively). The blood urea nitrogen (BUN):creatinine ratio had a positive correlation with serum pH (r = 0.16, p < 0.001). Twenty-nine admissions (6.8%) with a median age of 16 (13-17) years required imaging for altered mental status, and none of these patients were diagnosed with cerebral edema.

Conclusion: DKA is associated with noncompliance among pediatric patients, irrespective of their type of insurance. Markers of oxidative stress (WBC, platelets, and MPV) were associated with increased severity of DKA. The BUN:creatinine ratio may not provide accurate hydration status among DKA patients. Clinicians need to have a lower threshold for head imaging among younger patients.

## Introduction

Diabetes, a significant cause of morbidity and mortality, affects 463 million people worldwide and is estimated to increase to 578 million by 2030 [1]. According to 2019 research by the International Diabetes Federation (IDF), the United States ranks second worldwide in type 1 diabetes prevalence among youth, with 224 children per 100,000 population among those younger than 19 years [2].

Diabetic ketoacidosis (DKA) is a life-threatening emergency affecting patients with diabetes; it is mostly seen in those with type 1 diabetes, but it can also be seen in type 2 diabetes patients [3]. Szypowska and Skórka (2011) showed that 26% of pediatric new-onset type 1 diabetes patients presented with DKA [4]. Among pediatric patients with known type 1 diabetes, the incidence of DKA ranges from 6% to 8% per year [5]. Moreover, the pediatric DKA mortality rate ranges from 0.15% to 0.3% and is mostly due to cerebral edema [6,7]. This study is designed to evaluate the various risk factors and laboratory findings associated with the severity of DKA among pediatric patients.

## Materials and methods

The study was a retrospective chart review of the electronic medical records over a 3.5-year period between October 2017 and April 2021 in a 20-bed pediatric intensive care unit (PICU) in an academic center in South Alabama. Patients with DKA were identified based on the International Classification of Disease Code (10th version) ICD-10 (E10.10). The exclusion criteria include those who were >18 years of age and those with serum pH > 7.30.

The lab results shown were obtained on admission, and computed tomography (CT) scans were read by a board-certified radiologist. Research approval was obtained from the institutional review board at the University of South Alabama. The statistical analysis was performed using IBM Statistical Package for the Social Sciences (SPSS) software, v25.0 (IBM Corp., Armonk, NY).

The categorical variables were evaluated using logistic regression, and continuous variables were evaluated using the Mann-Whitney U test for nonparametric data analysis. The continuous variables were compared using Pearson's correlation, and the degree of correlation was evaluated with the coefficient of correlation (r). Weak correlation is suggested by the coefficient of correlation (r) 0 to 0.39, moderate correlation is suggested by r between 0.4 and 0.59, and a strong correlation has r between 0.6 and 1.

## Results

**Figure 1:**
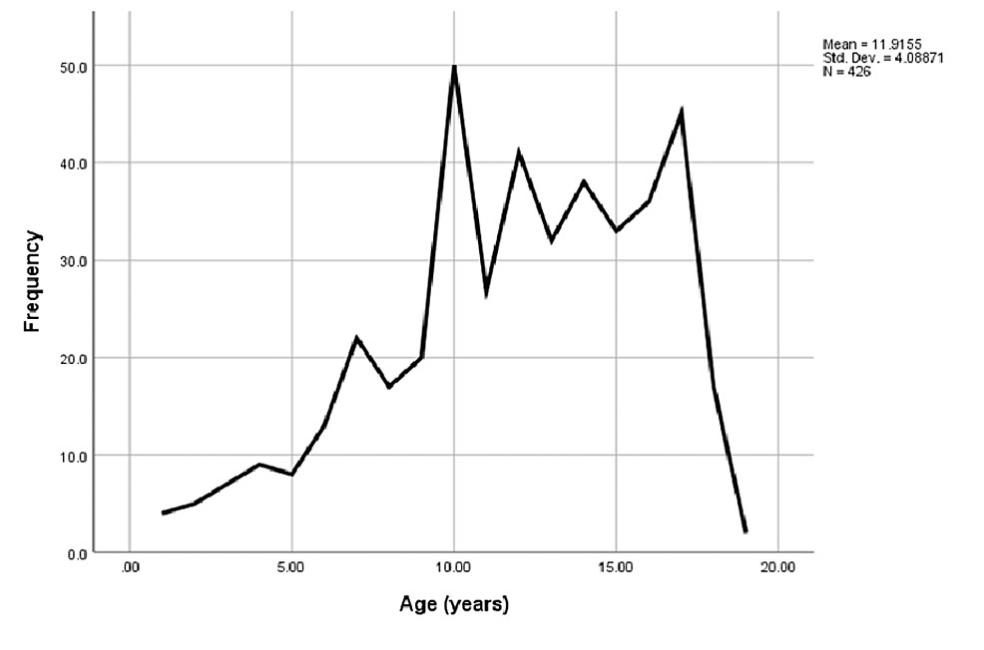
Age distribution of the patients presenting with DKA The image shows the age distribution of pediatric DKA patients with a mean age of 11.91 years and a standard deviation of 4.08 years. DKA: Diabetic ketoacidosis.

Of 429 patient encounters of those admitted with a diagnosis of DKA to the PICU during the 3.5-year period, there were 256 unique patients among which 55.9% of patients were males. The median age (interquartile range [IQR]) of the presenting patients was 12 (10-15) years (Figure 1). Age had a positive correlation with serum pH (r = 0.01, p < 0.001), and 27.7% of the patients who presented with DKA had private insurance. There was no difference in median (IQR) HbA1c level among the patients presenting with private insurance versus Medicaid (11 [9.65-12] vs 11.1 [9.87-12.2], p = 0.4, respectively). The median (IQR) length of stay (LOS) was two (two to three) days. Evaluation of body mass index (BMI) showed no significant correlation in terms of severity of DKA and serum pH (r = 0.027, p = 0.51). The median (IQR) serum pH of those who were admitted with DKA on admission was 7.17 (7.08-7.25) (Table 1).

**Table 1:** Laboratory findings on admission of the patients presented with DKA RBC: Red blood cells; WBC: White blood cells; MCV: Mean corpuscular volume; MCH: Mean cell hemoglobin; MCHC: Mean cell hemoglobin concentration; RDW: Red cell diameter width; RDW-SD: Red cell distribution width-standard deviation; MPV: Mean platelet volume; HbA1c: Hemoglobin A1c.

Variables	Median (IQR)
Venous PO_2_ (mmHg)	45 (35–58.25)
Venous PCO_2_ (mmHg)	29 (24–34)
Venous pH	7.17 (7.08–7.25)
Base deficit	-16.95 (-21.6–12.38)
Venous oxygen saturation (%)	75 (61–87)
Serum sodium level (mg/dl)	135 (131–138)
Serum potassium level (mg/dl)	4.4 (4–5)
Serum chloride (mg/dl)	103 (99–107.25)
Serum glucose (mg/dl)	379 (254–507)
Blood urea nitrogen (BUN) values (mg/dl)	16 (12–22)
Serum creatinine (mg/dl)	0.99 (0.8–1.24)
BUN:creatinine ratio	15.56 (11.89–20.33)
Urine protein (mg/dl)	30 (15–49.07)
Anion gap	20 (16–23.25)
Serum bicarbonate (mmol/L)	10 (7–14)
HbA1c	11.02 (10–12)
WBC (x 10^9^/L)	17.9 (10.99–19.98)
Hemoglobin (mg/dl)	13.54 (12.4–14.3)
RDW (%)	13.31 (12.7–13.6)
RDW-SD (fL)	40.61 (38.57–41.32)
RBC (x 10^9^/L)	5.1 (4.78–5.39)
Hematocrit	42.93 (40.1–45.6)
MCV	84.45 (81.6–86.9)
MCH	28.28 (27.8–29.3)
MCHC	33.53 (32.9–34.4)
Platelets (x 10^9^/L)	358.73 (298–298)
MPV	10.75 (10.3–11.1)

**Figure 2:**
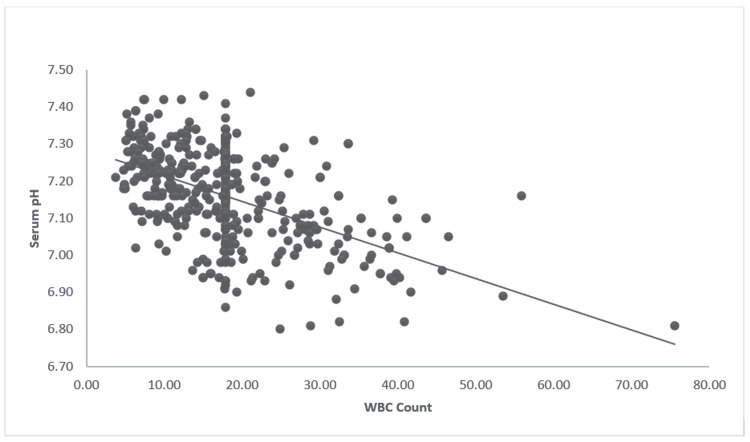
Correlation of the WBC and serum pH in DKA patients The image shows a scatter plot depicting correlation between WBC count and serum pH; r = -0.52, p < 0.001. WBC: White blood cell; DKA: Diabetic ketoacidosis.

The WBC count on admission was inversely proportional to serum pH (r = -0.52, p < 0.001) (Figure 2). Similarly, this study compared other variables to see their association with the severity of metabolic acidosis on presentation (Table 2).

**Table 2:** Linear regression of laboratory findings with serum pH as the dependent variable RBC: Red blood cells; WBC: White blood cells; MCV: Mean corpuscular volume; MCH: Mean cell hemoglobin; MCHC: Mean cell hemoglobin concentration; RDW: Red cell diameter width; RDW-SD: Red cell distribution width-standard deviation; MPV: Mean platelet volume; HbA1c: Hemoglobin A1c.

Variables	Standardized coefficients beta	Correlation coefficients	p-value
WBC count	-0.47	-0.52	<0.001
RBC count	0.88	-0.18	0.08
Platelet count	0.13	-0.25	0.01
Hemoglobin	0.08	0.06	0.24
Hematocrit	-1.10	-0.32	0.03
MCV	0.21	-0.23	0.72
MCH	0.17	-0.01	0.76
MCHC	-0.13	0.28	0.74
RDW	-0.17	-0.14	0.39
RDW-SD	0.04	-0.28	0.86
MPV	0.10	-0.11	0.03
HbA1c	-0.08	-0.05	0.04
Urine protein	-0.08	-0.09	0.03
Serum glucose	-0.21	-0.35	<0.001

**Figure 3:**
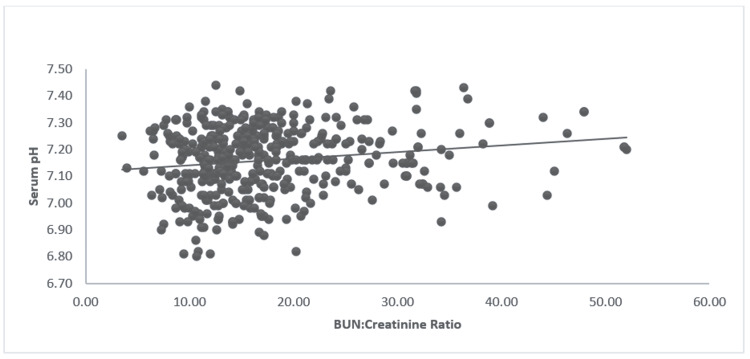
Correlation of BUN:creatinine ratio and serum pH The image shows a scatter plot depicting the correlation between BUN:creatinine ratio and serum pH; r = 0.16, p < 0.001. BUN: Blood urea nitrogen.

**Figure 4:**
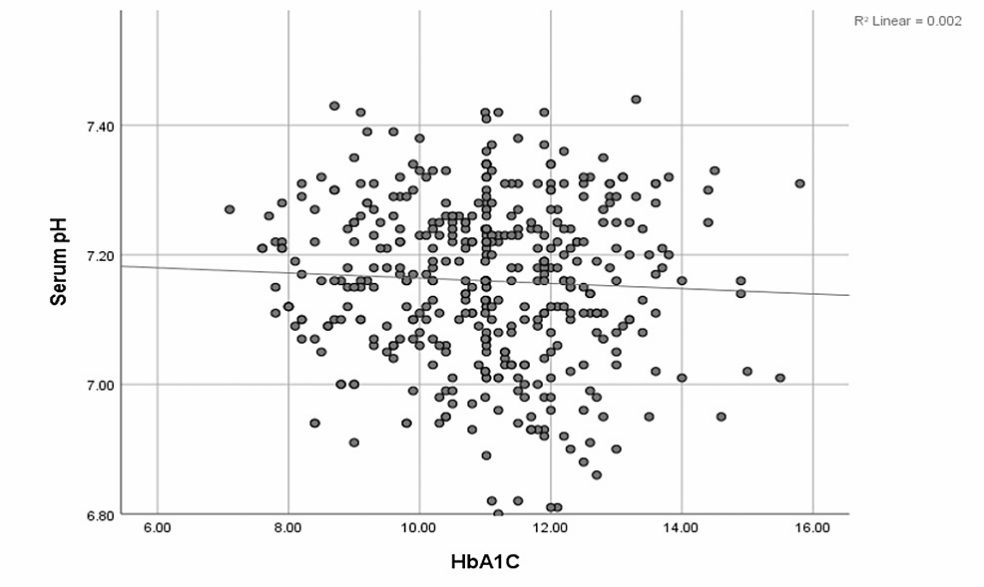
Correlation of the serum pH and HbA1c values in DKA patients The image shows a scatter plot depicting the correlation between WBC count and serum pH; r = -0.05, p = 0.04. WBC: White blood cell; DKA: Diabetic ketoacidosis; HbA1c: Hemoglobin A1c.

**Figure 5:**
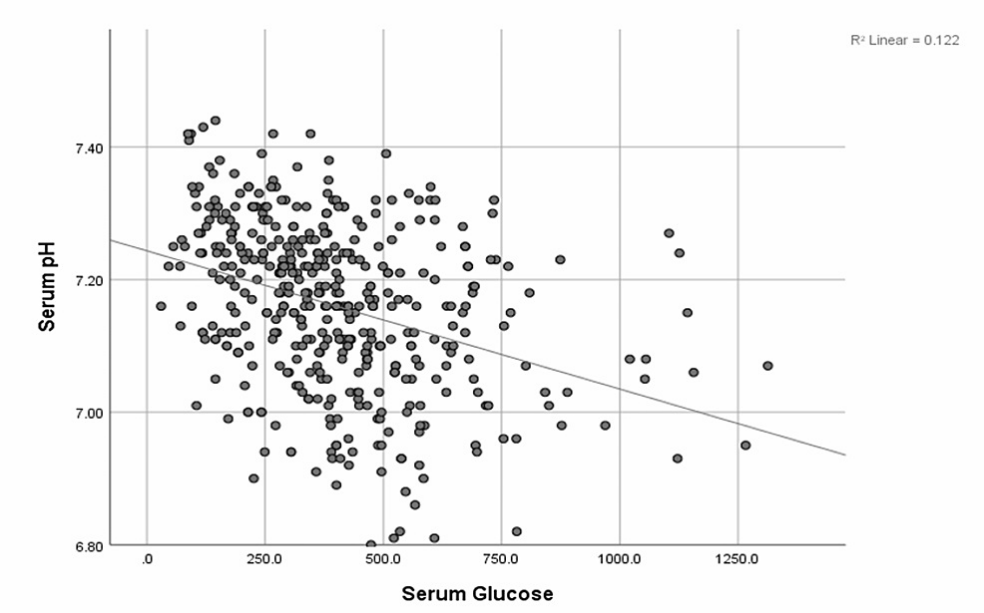
Correlation of blood glucose with serum pH values in DKA The image shows a scatter plot depicting the correlation between blood glucose with serum pH values in DKA; r = -0.35, p < 0.001. DKA: Diabetic ketoacidosis.

The median (IQR) serum pH was 7.17 (7.08-7.25). The blood urea nitrogen (BUN):creatinine ratio was associated with a positive correlation with serum pH (0.16, p < 0.001) (Figure 3), although neither BUN (-0.12, p = 0.42) nor serum creatinine (-0.27, p = 0.09) on admission was independently associated with changes in serum pH. While serum pH had a negative correlation with the BUN:creatinine ratio, HbA1c, and blood glucose levels (Figures 3-5).

The 29 patient encounters (6.8%) had a head CT scan, which was done for altered mental status at some point during their admission. The median (IQR) age of the patients who received a head CT scan was 16 (13-17) years, which was older than those who did not get a head CT scan: 12 (9-15) years (p < 0.001). There was no evidence of cerebral edema in any of these CT scans. Older patients were more likely to get head CT scans; OR 1.31 (1.1-1.55) (Table 3).

**Table 3:** Multivariate logistic regression evaluating the factors affecting the probability of getting radiological imaging for altered mental status among DKA patients DKA: Diabetic ketoacidosis; BUN: Blood urea nitrogen; HbA1c: hemoglobin A1c; LOS: Length of stay.

Variables	Odds ratio	p-value
Age	1.31 (1.1–1.55)	0.02
BUN	1.06 (1.01–1.11)	0.01
HbA1c	0.8 (0.59–1.08)	0.15
LOS	1.13 (0.9–1.42)	0.30
Serum glucose	1 (1–1)	0.71
Anion gap	1.07 (0.95–1.22)	0.26
pCO_2_	0.97 (0.87–1.09)	0.72
Serum sodium	1.02 (0.93–1.12)	0.73
BUN:creatinine ratio	1.01 (0.93–1.1)	0.78
Serum pH	4.25 (0.02–11551)	0.71

## Discussion

Szypowska et al. (2016) showed that DKA is more frequently seen in patients younger than five years [8]. Moreover, the current study showed that younger age at presentation was associated with severe metabolic acidosis as well, although a few studies have shown no correlation between the age and severity of DKA [9,10]. The difference could be explained by the fact that these previous studies had smaller sample sizes and focused on new-onset DKA episodes, whereas this study considered all DKA episodes.

The median age of patients admitted with DKA was 12 years (Figure 1), which was similar to other studies [11], although the patients in these studies focused on new-onset type 1 diabetes patients who were younger compared to those in our study [8]. Only 5.76% of hospitalized DKA patients were new-onset diabetes cases. This could be an underestimation of actual new-onset diabetes mellitus (DM) patients hospitalized as DKA patients since some of these new-onset DKA patients were hospitalized before the study duration period.

A majority of the patients (72.3%) who presented with DKA had either Medicaid or self-insurance policies, which could be related to the volume of Medicaid patients seen at the institution rather than noncompliance as evidenced by similar HbA1c levels recorded in both groups. Both groups had significantly higher HbA1c levels than desired, which emphasizes the role of compliance by patients in preventing DKA admissions (Table 1). Noncompliance contributes to increased unplanned readmissions among these patients [12].

CT head scans remain the most used radiological modality for addressing concerns of altered mental status among DKA patients. The risk factors that were significantly associated with a probability of obtaining radiological imaging for altered mental status were older age of the patients and elevated BUN (Table 3). Studies have shown that the risk factors associated with cerebral edema in DKA are age under five years, high BUN, low pCO_2_, administration of sodium bicarbonate, and degree of dehydration [6,13].

We did not find any correlation between HbA1c, serum glucose levels, severity of acidosis, and anion gap with the probability of obtaining head CT scans for altered mental status. This could be related to the ability of the adolescents to better communicate as compared to younger patients, thus leading to a better assessment of mental status. Therefore, the probability of obtaining a head CT scan may be higher among the older population, while there may be a higher probability of finding cerebral edema among younger patients.

Serum glucose at presentation had a much stronger correlation to the severity of acidosis than HbA1c levels (Figures 4, 5). Elevated glucose levels could be a combination of both noncompliance and stress, leading to a stronger correlation with the serum pH value [14]. Elevated BUN:creatinine ratio suggests prerenal dehydration; interestingly, patients who were more dehydrated were less likely to have severe acidosis (Figure 3). This may be related to earlier presentation by dehydrated patients compared to those who were able to maintain relatively better hydration. Another reason behind this observation could be falsely elevated serum creatinine levels due to elevated serum ketones [15,16]. Ugale et al. (2012) showed that the measured degree of dehydration does not correlate with the severity of DKA in pediatric patients [17]. Therefore, this study reiterates the fact that the BUN:creatinine ratio in DKA patients might not be the best indicator of patient dehydration and severity of DKA.

Both leukocytosis and thrombocytosis were associated with severe metabolic acidosis (Figure 2 and Table 2): This could be a stress response [18]. While WBC counts are more likely to be elevated for a few days after stress, maximal thrombocytosis can take up to two weeks after the onset of stress to be elevated [19]. This might indicate that the onset of stress among patients presenting with DKA might be earlier than commonly reported by most patients. While most DKA patients had a normal mean platelet volume (MPV), it was associated with increased severity of metabolic acidosis in DKA (Tables 1, 2).

Mousa et al. (2017) showed that oxidative stress in DKA leads to morphological changes in platelets that are often associated with increased MPV, which in our study was also found to be associated with increased severity of DKA [20]. This is an important distinction, especially in the setting of increasing incidence of pediatric sepsis [21].

Limitations

While the sample size of this study is comparable to other pediatric studies for DKA, it is a single-institution study; therefore, practices may vary compared to other centers. This could also pertain to the threshold for obtaining radiological imaging. Lab results were obtained at admission to maintain uniformity among the sample population, and although it would be beyond the scope of this study, it would be interesting to compare the trends of lab results throughout the rest of the hospitalization and to observe the effects of various clinical interventions.

The study uses the ICD-10 code of identification of DKA patients, which does not differentiate between the new-onset and known DKA patients (one of the risk factors of severity of illness); therefore, further studies taking this into account will provide more information. Additionally, the study does not divide the type 1 vs type 2 DM among those who presented with DKA, which can be something that can be studied separately. As inherent to most retrospective studies, the correlation between various variables cannot assume the causation, for which a randomized control study design is recommended.

## Conclusions

DKA continues to be a common cause of pediatric ICU admissions among adolescents. This study did not find any correlation between the BMI and severity of DKA. Significantly higher HbA1c values among those patients presenting with DKA suggest noncompliance as being an important factor, irrespective of the type of insurance held. The degree of oxidative stress response (elevated WBC, platelets, and MPV) correlates with the degree of metabolic acidosis on admission. Other factors associated with severe metabolic acidosis at presentation include elevated hematocrit, elevated HbA1c levels, elevated serum glucose levels, and elevated urine protein levels. The BUN:creatinine ratio did not correlate with the severity of illness, and it might not provide a good estimation of hydration status in DKA patients. Older patients are more likely to get a head CT scan for altered mental status, which does not correlate with cerebral edema findings, indicating that we probably need to have a lower threshold for obtaining imaging in younger patients.
